# Vibration Transmission Characteristics of Bistable Nonlinear Acoustic Metamaterials Based on Effective Negative Mass

**DOI:** 10.3390/nano15161269

**Published:** 2025-08-17

**Authors:** Ming Gao, Guodong Shang, Jing Guo, Lingfeng Xu, Guiju Fan

**Affiliations:** 1 Mechanical and Electronic Engineering College, Shandong Agricultural University, Tai’an 271018, China; gaom@sdau.edu.cn (M.G.); 13615383504@163.com (G.S.); gjcp@sdau.edu.cn (J.G.); 2 Shandong Engineering Research Center of Agricultural Equipment Intelligentization, Shandong Agricultural University, Tai’an 271018, China; 3 Shandong Higher Education Institution Future Industry Engineering Research Center of Intelligent Agricultural Robots, Shandong Agricultural University, Tai’an 271018, China; 4 Shandong Key Laboratory of Intelligent Production Technology and Equipment for Facility Horticulture, Shandong Agricultural University, Tai’an 271018, China

**Keywords:** nonlinear acoustic metamaterials, bistability, effective negative mass, vibration transmission characteristics, zero mass

## Abstract

The growing demand for low-frequency, broadband vibration and noise suppression technologies in next-generation mechanical equipment has become increasingly urgent. Effective negative mass locally resonant structures represent one of the most paradigmatic classes of acoustic metamaterials. Their unique elastic wave bandgaps enable efficient suppression of low-frequency vibrations, while inherent nonlinear effects provide significant potential for the design and tunability of these bandgaps. To achieve ultra-low-frequency and ultra-broadband vibration attenuation, this study employs Duffing oscillators exhibiting negative-stiffness characteristics as structural elements, establishing a bistable nonlinear acoustic-metamaterial mechanical model. Subsequently, based on the effective negative mass local resonance theory, the perturbation solution for the dispersion curves is derived using the perturbation method. Finally, the effects of mass ratio, stiffness ratio, and nonlinear term on the starting and cutoff frequencies of the bandgap are analyzed, and key geometric parameters influencing the design of ultra-low vibration reduction bandgaps are comprehensively investigated. Subsequently, the influence of external excitation amplitude and the nonlinear term on bandgap formation is analyzed using numerical computation methods. Finally, effective positive mass, negative mass, and zero-mass phenomena within distinct frequency ranges of the bandgap and passband are examined to validate the theoretically derived results. The findings demonstrate that, compared to a positive-stiffness system, the bandgap of the bistable nonlinear acoustic metamaterial incorporating negative-stiffness Duffing oscillators shifts to higher frequencies and widens by a factor of $\sqrt{2}$. The external excitation amplitude *F* changes the bandgap starting frequency and cutoff frequency. As *F* increases, the starting frequency rises while the cutoff frequency decreases, resulting in a narrowing of the bandgap width. Within the frequency range bounded by the bandgap starting frequency and cutoff frequency, the region between the resonance frequency and cutoff frequency corresponds to an effective negative mass state, whereas the region between the bandgap starting frequency and resonance frequency exhibits an effective positive mass state. Critically, the bandgap encompasses both effective positive mass and negative mass regions, wherein vibration propagation is suppressed. Concurrently, a zero-mass state emerges within this structure, with its frequency precisely coinciding with the bandgap cutoff frequency. This study provides a theoretical foundation and practical guidelines for designing nonlinear acoustic metamaterials targeting ultra-low-frequency and ultra-broadband vibration and noise mitigation.

## 1. Introduction

With the widespread application of precision instruments in aerospace, micro–nano manufacturing, high-precision measurement, and other fields, the requirements for environmental vibration and noise control have become increasingly stringent. Without effective vibration-isolation materials, low-frequency vibrations and noise from the environment can be transmitted directly to precision equipment, leading to a series of adverse consequences. In micro–nano manufacturing, vibrational disturbances can introduce machining errors, leading to increased surface roughness and dimensional deviations, which in turn reduce product yield. Furthermore, prolonged exposure to vibrations can induce fatigue damage in mechanical components, shortening equipment lifespan and increasing maintenance costs. Additionally, vibration-induced noise not only degrades equipment performance but may also adversely affect the working environment of operators, causing auditory fatigue and even occupational health risks. Traditional vibration-isolation materials perform well in the mid-to-high-frequency range, but they struggle to achieve both lightweight design and effective low-frequency vibration isolation. Acoustic metamaterials, a novel class of engineered functional materials that manipulate macroscopic properties through artificially designed unit structures, have attracted significant attention in both academia and engineering in recent years. This is due to their ability to generate bandgap effects within specific frequency ranges, thereby effectively blocking acoustic wave propagation and vibration transmission. These materials provide new design principles and technical approaches for low-frequency vibration suppression in precision instruments.

Acoustic metamaterials [1] represent a class of artificially engineered structures or composite materials designed to achieve extraordinary acoustic properties. The most prevalent types include locally resonant acoustic metamaterials [2,3], Helmholtz resonator-based acoustic metamaterials [4], and gradient-index acoustic metamaterials [5]. These materials can exhibit unconventional acoustic characteristics within specific frequency ranges that are unattainable in natural materials, thereby overcoming the physical limitations of conventional acoustic materials and enabling exceptional manipulation of sound waves. Currently developed acoustic metamaterials have demonstrated remarkable vibration-isolation capabilities [6,7,8]. Beyond vibration isolation, they exhibit other unique physical properties such as negative stiffness [9,10], negative mass density [11,12], negative elastic modulus [13], negative Poisson’s ratio [14,15], and bandgap characteristics [16,17]. Their fundamental principle lies in transcending the physical constraints of natural materials through sub-wavelength structural designs that enable extraordinary control over acoustic wave propagation. Unlike conventional materials whose properties depend on chemical composition, these metamaterials manipulate sound and vibration through precisely engineered architectures, granting them distinctive advantages in mechanical vibration and noise control. Recent years have witnessed significant advancements in the research and application of metamaterials, with expanding potential applications. By designing specialized acoustic structures, these materials can effectively control vibrations and noise. Gao, Ming et al. [18] constructed and analyzed a locally resonant unit incorporating a pure Duffing oscillator. By introducing a nonlinear term, they investigated its influence on the acoustic bandgap. Their study revealed that positive effective mass can also attenuate vibrations within the low-frequency bandgap region, thereby supplementing prior research that focused solely on negative effective mass. This finding challenges the conventional singular perspective that “negative mass = bandgap”, offering a more nuanced understanding of vibro-acoustic attenuation mechanisms. Wu, Yu dong et al. [19] proposed a lightweight plate-type locally resonant acoustic metamaterial to mitigate low-frequency vehicular noise and derived a design methodology for its low-frequency flexural wave bandgap. This approach effectively reduced low-frequency noise during both acceleration and constant-speed driving conditions. Chen et al. [20] achieved sound insulation using a novel hexagonal Helmholtz resonator design. Their numerical and experimental studies on both unit cells and the complete acoustic-metamaterial cavity demonstrated the reliability and feasibility of the proposed metastructure. By reconfiguring the cavity arrangement, the device maintained high insertion loss while exhibiting improved ventilation capacity. This highly tunable system enables active control for simultaneous noise cancelation and unimpeded airflow, making it suitable for various practical applications. Gai, Xiao-Ling et al. [21] proposed a Helmholtz-type honeycomb sandwich acoustic metamaterial. Through impedance tube experiments, they compared the sound absorption and insulation performance among the honeycomb panel, perforated honeycomb panel, and this metamaterial structure. The results demonstrate that this structure exhibits superior acoustic performance over a broad frequency range compared to the other two configurations, thereby enhancing the acoustic properties of honeycomb sandwich structures. Pan et al. [22] developed a three-dimensional polygonal double-negative (PDN) metamaterial exhibiting both negative stiffness and negative Poisson’s ratio. Tao et al. [23] proposed an acoustic-metamaterial plate based on negative Poisson’s ratio structures. Compared with conventional designs, their novel configuration achieved lower-frequency bandgaps and broader bandwidth. These findings expand the design possibilities for low-frequency broadband acoustic devices in dynamic environments while providing new methodologies for real-time bandgap tuning.

The introduction of nonlinearity can serve as an effective approach to enhance the performance of acoustic metamaterials. Sheng et al. [24] designed a high-stiffness nonlinear acoustic-metamaterial honeycomb sandwich panel and experimentally demonstrated that adding only a small additional mass significantly suppressed all resonances below 800 Hz. Hu et al. [25] investigated the propagation and mitigation of shock waves in a nonlinear metamaterial beam. Their structure achieved remarkable attenuation of impact-induced vibrations, outperforming its linear counterpart. This finding may inspire further research and applications. Fang et al. [26] systematically studied the vibration and acoustic radiation of nonlinear acoustic-metamaterial plates using both experimental and theoretical methods. Their work elucidated the modulation of nonlinear resonant mode amplitudes and shapes, providing critical insights into the design, fabrication, and application of nonlinear metamaterials. Negative stiffness Duffing oscillators [27,28] exhibit pronounced nonlinear characteristics and are widely employed in nonlinear vibration absorbers, particularly for handling large-amplitude vibrations or complex loading conditions. These systems also demonstrate bistable behavior [29,30], which can alter the equivalent stiffness or mass distribution of the material, thereby enabling further tuning of bandgaps in acoustic wave propagation [31]. Incorporating negative-stiffness elements into metamaterials offers significant advantages in vibration control, demonstrating great potential for vibration isolation. Vibration systems with negative-stiffness oscillators are characterized by low natural frequencies, high load-bearing capacity, and superior damping performance. Consequently, negative-stiffness mechanisms have seen increasing applications in vibration suppression in recent years. Li et al. [32] investigated the influence of negative-stiffness nonlinearity on the performance of a tuned negative-stiffness inerter damper (TNSID) and proposed a novel optimization method for its structural parameters, enhancing its vibration-control effectiveness. Barredo et al. [33] introduced an optimized design for a negative-stiffness-based nonlinear inerter dynamic vibration absorber (NS-NIDVA). This absorber reduced the dynamic amplification factor by approximately 50% and expanded the effective working bandwidth from 46% to 82%, demonstrating superior dynamic performance compared to other modern damping systems. Ji et al. [34] developed a negative-stiffness isolator based on a displacement amplification mechanism, tripling the linear negative-stiffness region. Conventional magnetic negative-stiffness mechanisms are often limited by excessive nonlinearity and bulky dimensions. To address this issue, Wu et al. [35] proposed a novel compact arrayed magnetic-spring negative-stiffness structure. The proposed design achieved high negative-stiffness density, effectively lowering the resonance frequency and broadening the vibration-isolation bandwidth, with analytical results showing good agreement with experiments. Chowdhury et al. [36] introduced a negative-stiffness inerter-based passive damper along with its optimal design parameters, offering a cost-effective solution with outstanding vibration suppression performance. Vo et al. [37] revisited the spring-mass model for metamaterials/metastructures, re-examining the formation of bandgaps in metal–concrete and metal–truss structures associated with effective negative mass and stiffness, and identified the fundamental mechanisms governing bandgap formation.

While most of the existing literature focuses on the bandgap behavior of acoustic metamaterials, studies concerning passband characteristics and the phenomenon of zero mass remain limited. To address this gap, the present study first reviews the dynamic models of metamaterials proposed in Refs. [18,38]. Building upon these frameworks, we develop a novel acoustic-metamaterial model incorporating negative stiffness. The structure consists of a rigid rod with an external cavity, connected to an internal Duffing oscillator of mass *m* via two identical springs of stiffness −k, both assumed to be massless. By introducing a Duffing oscillator—characterized by its unique nonlinear dynamic properties—into the metamaterial system and applying a harmonic excitation force, numerical simulations conducted in MATLAB R2023b demonstrate that the passband range can be dynamically tuned by adjusting the amplitude of the excitation. This finding reveals the potential for real-time adaptive control of the passband, enabling selective transmission of target frequencies while effectively suppressing unwanted vibrations. Furthermore, the existence of the zero-mass phenomenon is confirmed through numerical validation. At the zero-mass point, the system reaches a stable state. Specifically, the five-period metamaterial system incorporating negative-stiffness Duffing oscillators and exhibiting effective negative mass operates stably at this point, indicating that under such specific conditions, the system avoids chaotic, divergent, or unstable behavior and maintains favorable dynamic stability. This stability is crucial for ensuring the reliability and practicality of system performance in real-world applications, highlighting the significance of the proposed model for the future development of acoustic metamaterials. The remainder of this paper is organized as follows: Section 2 presents the theoretical analysis of a periodic structure composed of negative-stiffness Duffing oscillators. Through force analysis, the dispersion relation is derived, and the first-order perturbation solutions for the acoustic and optical branches are obtained using the perturbation method. The starting and cutoff frequencies of the bandgap are then calculated, and the influence of the stiffness ratio and mass ratio on the starting frequency is analyzed in the MATLAB environment. Section 3 aims to validate the theoretical analysis by establishing the equations of motion for a five-period system with negative-stiffness Duffing oscillators based on Newton’s second law. Numerical simulations in MATLAB are conducted to examine the effects of shell stiffness and external excitation amplitude F on the bandgap width. The results confirm the existence of both effective positive and negative mass regions and further verify the occurrence of the zero-mass phenomenon, consistent with theoretical predictions. Finally, Section 4 summarizes the main findings and conclusions of this study.

## 2. First-Order Regenerative Solutions of Acoustic Metamaterial Dispersion Curves for Duffing Oscillators Containing Negative Stiffness

**Figure 1:**
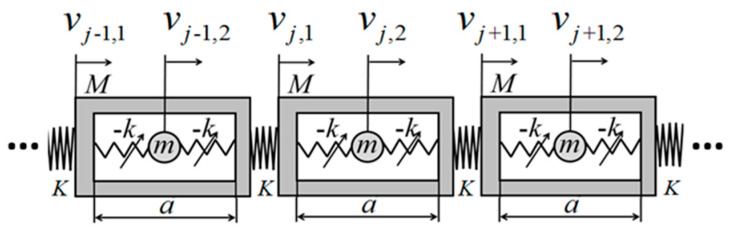
Periodic structure of effective negative mass with negative-stiffness Duffing oscillator.

Inspired by the metamaterial models proposed in Refs. [18,38], a metamaterial model incorporating negative-stiffness characteristics is established. An infinitely periodic metamaterial configuration, as depicted in Figure 1, is considered, with a shell length of a, with an outer shell mass of M, a spring stiffness factor of K connecting the two outer shells, an inner oscillator mass of m, an inner oscillator that is a negative-stiffness Duffing oscillator, a spring linear stiffness factor of −K connecting the negative-stiffness Duffing oscillator to the outer shell, and a coefficient of Γ in front of the cubic term of the displacement. And for the jst period, $u_{j,1}$ and $u_{j,2}$ denote the displacements of the shell and the negative-stiffness Duffing oscillator for the jth period, respectively.$u_{j-1,1}$ and $u_{j-1,2}$ denote the displacements of the shell and the negative-stiffness Duffing oscillator for the j−1th period, respectively. And $u_{j+1,1}$ and $u_{j+1,2}$ denote the displacements of the shell and the negative-stiffness Duffing vibrator for the j+1th period, respectively. The system is modeled dynamically in the following figure.

From the force characteristics of the shell in the jst period, it is not only subjected to the forces $f_{1}$ and $f_{2}$ given to it by the internal negative-stiffness Duffing oscillator, but also subjected to the forces applied to it by the shell in the j+1th and j−1th periods, and its differential equations of motion are established as follows:

(1) $$M{\ddot{u}}_{j,1}-f_{1}-f_{2}+K(2u_{j,1}-u_{j-1,1}-u_{j+1,1})=0$$

And according to the force on the negative-stiffness Duffing oscillator in the jst period, it is only subjected to the reaction forces $-f_{1}$ and $-f_{2}$ given to it by the shell in the jnd period, and its differential equation of motion is

(2) $$m{\ddot{u}}_{j,2}=-f_{1}-f_{2}=2k(u_{j,2}-u_{j,1})-2\varepsilon \Gamma {\left(u_{j,2}-u_{j,1}\right)}^{3}$$

where ε is a small amount of nonlinear perturbation much smaller than 1.

Join (1) and (2), eliminating $f_{1}$ and $f_{2}$, and the formula in Ref. [18]:

(3) $$M{\ddot{u}}_{j,1}+K(2u_{j,1}-u_{j-1,1}-u_{j+1,1})+2k(u_{j,2}-u_{j,1})-2\varepsilon \Gamma {\left(u_{j,2}-u_{j,1}\right)}^{3}=0$$

### 2.1. Solve for the Equilibrium Point

The equilibrium points of the shell and the internal negative-stiffness Duffing oscillator are found below:

For Equation (2), $2k(u_{j,2}-u_{j,1})-2\Gamma {\left(u_{j,2}-u_{j,1}\right)}^{3}=0$, available

(4) $$(u_{j,2}-u_{j,1})(k-\Gamma {\left(u_{j,2}-u_{j,1}\right)}^{2})=0$$

$u_{j,2}-u_{j,1}=0$ or $u_{j,2}-u_{j,1}=\pm \sqrt{\frac{k}{\Gamma }}$.

For Equation (3), $2k(u_{j,2}-u_{j,1})-2\Gamma {\left(u_{j,2}-u_{j,1}\right)}^{3}=0$, then the following can be obtained:

(5) $$K(2u_{j,1}-u_{j-1,1}-u_{j+1,1})=0$$

i.e., $u_{j,1}=0$.

From the above, the vibration of the shell has one equilibrium point $u_{j,1}=0$, and the internal negative-stiffness Duffing vibrator has three equilibrium points $u_{j,2}=0,\pm \sqrt{\frac{k}{\Gamma }}$, where $u_{j,2}=0$ is the unstable equilibrium point and $u_{j,2}=\pm \sqrt{\frac{k}{\Gamma }}$ is the stable equilibrium point. The following coordinate transformation is used:

(6) $$\left\{\begin{array}{c} u_{j,1}=v_{j,1}\\ u_{j,2}=v_{j,2}+\sqrt{\frac{k}{\Gamma }} \end{array}\right.$$

Substitute Equation (6) into Equations (2) and (3) write them in matrix form:

(7) $$\begin{array}{l} \left[\begin{array}{cc} M & 0\\ 0 & m \end{array}\right]\left[\begin{array}{c} {\ddot{v}}_{j,1}\\ {\ddot{v}}_{j,2} \end{array}\right]+\left[\begin{array}{cc} 2K+4k & -4k\\ -4k & 4k \end{array}\right]\left[\begin{array}{c} v_{j,1}\\ v_{j,2} \end{array}\right]-\left[\begin{array}{c} K(v_{j-1,1}+v_{j+1,1})\\ 0 \end{array}\right]\\ +2\varepsilon \Gamma \left[\begin{array}{c} -{\left(v_{j,2}-v_{j,1}\right)}^{3}+3\sqrt{\frac{k}{\Gamma }}{\left(v_{j,2}-v_{j,1}\right)}^{2}\\ {\left(v_{j,2}-v_{j,1}\right)}^{3}-3\sqrt{\frac{k}{\Gamma }}{\left(v_{j,2}-v_{j,1}\right)}^{2} \end{array}\right]=0 \end{array}$$

Here, the linear stiffness of the spring of the shell and the negative-stiffness Duffing oscillator is defined as $\alpha =\frac{K}{k}$, the mass ratio as $\beta =\frac{M}{m}$, the linear intrinsic frequency of the negative-stiffness Duffing oscillator as $\omega_{n}=\sqrt{\frac{2k}{m}}$, and the introduction of the dimensionless time as τ=ωt, and the dimensionless nonlinear coefficients as $\bar{\Gamma }=\frac{\Gamma }{m\omega_{n}^{2}}$, which can be used as dimensionless Equation (7)

(8) $$\begin{array}{l} {\bar{\omega }}^{2}\left[\begin{array}{cc} \beta & 0\\ 0 & 1 \end{array}\right]\left[\begin{array}{c} \frac{d^{2}v_{j,1}}{d\tau^{2}}\\ \frac{d^{2}v_{j,2}}{d\tau^{2}} \end{array}\right]+\left[\begin{array}{cc} 2+\alpha & -2\\ -2 & 2 \end{array}\right]\left[\begin{array}{c} v_{j,1}\\ v_{j,2} \end{array}\right]-\frac{\alpha }{2}\left[\begin{array}{c} v_{j-1,1}+v_{j+1,1}\\ 0 \end{array}\right]\\ \;+2\varepsilon \bar{\Gamma }\left[\begin{array}{c} -{\left(v_{j,2}-v_{j,1}\right)}^{3}+3\sqrt{\frac{k}{\Gamma }}{\left(v_{j,2}-v_{j,1}\right)}^{2}\\ {\left(v_{j,2}-v_{j,1}\right)}^{3}-3\sqrt{\frac{k}{\Gamma }}{\left(v_{j,2}-v_{j,1}\right)}^{2} \end{array}\right]=0 \end{array}$$

where $\bar{\omega }=\frac{\omega }{\omega_{n}}$ is a dimensionless frequency. The first-order regenerative solution of $\bar{\omega }$ is solved below using the regenerative method.

Utilize the following progressive unfolding:

(9) $$\left\{\begin{array}{c} v_{j}=v_{j}^{\left(0\right)}+\varepsilon v_{j}^{\left(1\right)}+o(\varepsilon^{2})\\ \bar{\omega }={\bar{\omega }}_{0}+\varepsilon {\bar{\omega }}_{1}+o(\varepsilon^{2}) \end{array}\right.$$

Substitute (9) into Equation (8):

(10) $$\begin{array}{l} {\left({\bar{\omega }}_{0}+\varepsilon {\bar{\omega }}_{1}\right)}^{2}\left[\begin{array}{cc} \beta & 0\\ 0 & 1 \end{array}\right]\left[\begin{array}{c} \frac{d^{2}v_{j,1}^{\left(0\right)}}{d\tau^{2}}+\varepsilon \frac{d^{2}v_{j,1}^{\left(1\right)}}{d\tau^{2}}\\ \frac{d^{2}v_{j,2}^{\left(0\right)}}{d\tau^{2}}+\varepsilon \frac{d^{2}v_{j,2}^{\left(1\right)}}{d\tau^{2}} \end{array}\right]+\left[\begin{array}{cc} 2+\alpha & -2\\ -2 & 2 \end{array}\right]\left[\begin{array}{c} v_{j,1}^{\left(0\right)}+\varepsilon v_{j,1}^{\left(1\right)}\\ v_{j,2}^{\left(0\right)}+\varepsilon v_{j,2}^{\left(1\right)} \end{array}\right]\\ -\frac{\alpha }{2}\left[\begin{array}{c} v_{j-1,1}^{\left(0\right)}+v_{j+1,1}^{\left(0\right)}+\varepsilon (v_{j-1,1}^{\left(1\right)}+v_{j+1,1}^{\left(1\right)})\\ 0 \end{array}\right]+2\varepsilon \bar{\Gamma }\left[\begin{array}{c} -{\left(v_{j,2}-v_{j,1}\right)}^{3}+3\sqrt{\frac{k}{\Gamma }}{\left(v_{j,2}-v_{j,1}\right)}^{2}\\ {\left(v_{j,2}-v_{j,1}\right)}^{3}-3\sqrt{\frac{k}{\Gamma }}{\left(v_{j,2}-v_{j,1}\right)}^{2} \end{array}\right]+o(\varepsilon^{2})=0 \end{array}$$

Separating the coefficients of $\varepsilon^{0}$ and $\varepsilon^{1}$ making them zero gives [18] as follows:

$\varepsilon^{0}$ coefficient:

(11) $${\bar{\omega }}_{0}^{2}\left[\begin{array}{cc} \beta & 0\\ 0 & 1 \end{array}\right]\left[\begin{array}{c} \frac{d^{2}v_{j,1}^{\left(0\right)}}{d\tau^{2}}\\ \frac{d^{2}v_{j,2}^{\left(0\right)}}{d\tau^{2}} \end{array}\right]+\left[\begin{array}{cc} 2+\alpha & -2\\ -2 & 2 \end{array}\right]\left[\begin{array}{c} v_{j,1}^{\left(0\right)}\\ v_{j,2}^{\left(0\right)} \end{array}\right]-\frac{\alpha }{2}\left[\begin{array}{c} v_{j-1,1}^{\left(0\right)}+v_{j+1,1}^{\left(0\right)}\\ 0 \end{array}\right]=0$$

$\varepsilon^{1}$ coefficient:

(12) $${\bar{\omega }}_{0}^{2}\left[\begin{array}{cc} \beta & 0\\ 0 & 1 \end{array}\right]\left[\begin{array}{c} \frac{d^{2}v_{j,1}^{\left(1\right)}}{d\tau^{2}}\\ \frac{d^{2}v_{j,2}^{\left(1\right)}}{d\tau^{2}} \end{array}\right]+\left[\begin{array}{cc} 2+\alpha & -2\\ -2 & 2 \end{array}\right]\left[\begin{array}{c} v_{j,1}^{\left(1\right)}\\ v_{j,2}^{\left(1\right)} \end{array}\right]-\frac{\alpha }{2}\left[\begin{array}{c} v_{j-1,1}^{\left(1\right)}+v_{j+1,1}^{\left(1\right)}\\ 0 \end{array}\right]\;=-2{\bar{\omega }}_{0}{\bar{\omega }}_{1}\left[\begin{array}{cc} \beta & 0\\ 0 & 1 \end{array}\right]\left[\begin{array}{c} \frac{d^{2}v_{j,1}^{\left(0\right)}}{d\tau^{2}}\\ \frac{d^{2}v_{j,2}^{\left(0\right)}}{d\tau^{2}} \end{array}\right]-2\bar{\Gamma }\left[\begin{array}{c} -{\left(v_{j,2}-v_{j,1}\right)}^{3}+3\sqrt{\frac{k}{\Gamma }}{\left(v_{j,2}-v_{j,1}\right)}^{2}\\ {\left(v_{j,2}-v_{j,1}\right)}^{3}-3\sqrt{\frac{k}{\Gamma }}{\left(v_{j,2}-v_{j,1}\right)}^{2} \end{array}\right]$$

### 2.2. Linear Dispersion Curve Equation

The steady-state solution of Equation (11) takes the following form:

(13) $$\left[\begin{array}{c} v_{j,1}^{\left(0\right)}\\ v_{j,2}^{\left(0\right)} \end{array}\right]=\left[\begin{array}{c} A_{1}^{\left(0\right)}\\ A_{2}^{\left(0\right)} \end{array}\right]\frac{1}{2}e^{iqaj}e^{i\tau }+c.c.$$

(14) $$\left[\begin{array}{c} v_{j\pm 1,1}^{\left(0\right)}\\ v_{j\pm 1,2}^{\left(0\right)} \end{array}\right]=\left[\begin{array}{c} A_{1}^{\left(0\right)}\\ A_{2}^{\left(0\right)} \end{array}\right]\frac{1}{2}e^{iqa(j\pm 1)}e^{i\tau }+c.c.$$

where a is the periodic unit size, qaj is the j periodic phase factor, and $A_{1}^{\left(0\right)}A_{2}^{\left(0\right)}$ are the steady-state amplitude of shell and oscillator, respectively. c.c. stands for the conjugation of the equations above.

Substitute (13) and (14) into Equation (11):

(15) $$\left[\begin{array}{cc} -{\bar{\omega }}_{0}^{2}\beta +2+2\alpha \sin^{2}(\frac{qa}{2}) & -2\\ -2 & 2-{\bar{\omega }}_{0}^{2} \end{array}\right]\left[\begin{array}{c} A_{1}^{\left(0\right)}\\ A_{2}^{\left(0\right)} \end{array}\right]\frac{1}{2}e^{iqaj}e^{i\tau }+c.c.=0$$

For Equation (15) to have a nonzero solution $A_{1}^{\left(0\right)}$$A_{2}^{\left(0\right)}$, the determinant of its coefficient matrix should be equal to zero, thus

(16) $$\left(\beta +\frac{2}{2-{\bar{\omega }}_{0}^{2}}){\bar{\omega }}_{0}^{2}=2\alpha \sin^{2}(\frac{qa}{2}\right)$$

Moreover, $A_{1}^{\left(0\right)}$ and $A_{2}^{\left(0\right)}$ have the following relational equation:

(17) $$A_{1}^{\left(0\right)}=(1-\frac{{\bar{\omega }}_{0}^{2}}{2})A_{2}^{\left(0\right)}$$

Solving for ${\bar{\omega }}_{0}$ in Equation (15) yields the dispersion curve equations for the two frequency branches [18]:

(18) $${\bar{\omega }}_{0}^{aco}=\frac{\sqrt{\beta [\beta +1+\alpha \sin^{2}(\frac{qa}{2})-\sqrt{{\left(\beta +1\right)}^{2}+\alpha \sin^{2}(\frac{qa}{2})(\alpha \sin^{2}(\frac{qa}{2})-2\beta +2)}]}}{\beta }$$

(19) $${\bar{\omega }}_{0}^{opt}=\frac{\sqrt{\beta [\beta +1+\alpha \sin^{2}(\frac{qa}{2})+\sqrt{{\left(\beta +1\right)}^{2}+\alpha \sin^{2}(\frac{qa}{2})(\alpha \sin^{2}(\frac{qa}{2})-2\beta +2)}]}}{\beta }$$

where ${\bar{\omega }}_{0}^{aco}$ and ${\bar{\omega }}_{0}^{opt}$ represent the acoustic frequency branch and the optical frequency branch, respectively. The acoustic frequency branch is a low-frequency dispersion curve and the optical frequency branch is a high-frequency dispersion curve.

### 2.3. Nonlinear Dispersion Curve Equation

Consider below the first-order regenerative solution, which for Equation (12) takes the following form:

(20) $$\left[\begin{array}{c} v_{j,1}^{\left(1\right)}\\ v_{j,2}^{\left(1\right)} \end{array}\right]=\left[\begin{array}{c} A_{1}^{\left(1\right)}\\ A_{2}^{\left(1\right)} \end{array}\right]\frac{1}{2}e^{iqaj}e^{i\tau }+c.c.$$

(21) $$\left[\begin{array}{c} v_{j\pm 1,1}^{\left(1\right)}\\ v_{j\pm 1,2}^{\left(1\right)} \end{array}\right]=\left[\begin{array}{c} A_{1}^{\left(1\right)}\\ A_{2}^{\left(1\right)} \end{array}\right]\frac{1}{2}e^{iqa(j\pm 1)}e^{i\tau }+c.c.$$

Substitute Equations (20) and (21) into (12):

(22) $$\begin{array}{l} \left[\begin{array}{cc} -{\bar{\omega }}_{0}^{2}\beta +2+2\alpha \sin^{2}(\frac{qa}{2}) & -2\\ -2 & 2-{\bar{\omega }}_{0}^{2} \end{array}\right]\left[\begin{array}{c} A_{1}^{\left(1\right)}\\ A_{2}^{\left(1\right)} \end{array}\right]\frac{1}{2}e^{iqaj}e^{i\tau }+c.c.=\left[\begin{array}{c} 2{\bar{\omega }}_{0}{\bar{\omega }}_{1}\beta A_{1}^{\left(0\right)}+4\bar{\Gamma }N^{\left(0\right)}\\ 2{\bar{\omega }}_{0}{\bar{\omega }}_{1}A_{2}^{\left(0\right)}-4\bar{\Gamma }N^{\left(0\right)} \end{array}\right]\frac{1}{2}e^{iqaj}e^{i\tau }\\ +\left[\begin{array}{c} A_{1}^{\left(0\right)}\\ A_{2}^{\left(0\right)} \end{array}\right]Ce^{2iqaj}e^{2i\tau }+\left[\begin{array}{c} A_{1}^{\left(0\right)}\\ A_{2}^{\left(0\right)} \end{array}\right]Ce^{3iqaj}e^{3i\tau }+c.c.+3\sqrt{\frac{k}{\Gamma }}({\bar{A}}_{1}^{\left(0\right)}A_{1}^{\left(0\right)}-{\bar{A}}_{2}^{\left(0\right)}A_{1}^{\left(0\right)}-{\bar{A}}_{1}^{\left(0\right)}A_{2}^{\left(0\right)}+{\bar{A}}_{2}^{\left(0\right)}A_{2}^{\left(0\right)}) \end{array}$$

Of these, $N^{\left(0\right)}=\frac{3}{4}({\bar{A}}_{2}^{\left(0\right)}-{\bar{A}}_{1}^{\left(0\right)}){\left(A_{1}^{\left(0\right)}-A_{2}^{\left(0\right)}\right)}^{2}$.

Considering only the equality between the $e^{iqaj}e^{i\tau }$ coefficients on both sides of Equation (22). Then, we have

(23) $$\left[\begin{array}{cc} -{\bar{\omega }}_{0}^{2}\beta +2+2\alpha \sin^{2}(\frac{qa}{2}) & -2\\ -2 & 2-{\bar{\omega }}_{0}^{2} \end{array}\right]\left[\begin{array}{c} A_{1}^{\left(1\right)}\\ A_{2}^{\left(1\right)} \end{array}\right]=\left[\begin{array}{c} 2{\bar{\omega }}_{0}{\bar{\omega }}_{1}\beta A_{1}^{\left(0\right)}+4\bar{\Gamma }N^{\left(0\right)}\\ 2{\bar{\omega }}_{0}{\bar{\omega }}_{1}A_{2}^{\left(0\right)}-4\bar{\Gamma }N^{\left(0\right)} \end{array}\right]$$

Let $C_{1}=-\beta {\bar{\omega }}_{0}^{2}+2+2\alpha \sin^{2}(\frac{qa}{2})$, consider Equation (17) and get

(24) $$C_{1}=\frac{4}{2-{\bar{\omega }}_{0}^{2}}$$

The augmented matrix of Equation (23) is linearly transformed and the following can be obtained:

$$\left(\begin{array}{ccc} C_{1} & -2 & 2{\bar{\omega }}_{0}{\bar{\omega }}_{1}A_{1}^{\left(0\right)}+4\bar{\Gamma }N^{\left(0\right)}\\ 0 & 0 & (2{\bar{\omega }}_{0}{\bar{\omega }}_{1}A_{2}^{\left(0\right)}-4\bar{\Gamma }N^{\left(0\right)})\frac{C_{1}}{2}-2{\bar{\omega }}_{0}{\bar{\omega }}_{1}\beta A_{1}^{\left(0\right)}+4\bar{\Gamma }N^{\left(0\right)} \end{array}\right)$$

For Equation (23) to have a nonzero solution $A_{1}^{\left(1\right)}$$A_{2}^{\left(1\right)}$, the rank of its coefficient matrix should be the same as the rank of its augmentation matrix, thus

(25) $$(2{\bar{\omega }}_{0}{\bar{\omega }}_{1}A_{2}^{\left(0\right)}-4\bar{\Gamma }N^{\left(0\right)})\frac{C_{1}}{2}+2{\bar{\omega }}_{0}{\bar{\omega }}_{1}\beta A_{1}^{\left(0\right)}+4\bar{\Gamma }N^{\left(0\right)}=0$$

solve for

(26) $${\bar{\omega }}_{1}=\frac{\bar{\Gamma }N^{\left(0\right)}{\bar{\omega }}_{0}}{2A_{2}^{\left(0\right)}+A_{1}^{\left(0\right)}\beta (2-{\bar{\omega }}_{0}^{2})}$$

Therefore, the first-order regenerative solution is of the form

(27) $$\bar{\omega }={\bar{\omega }}_{0}+\varepsilon {\bar{\omega }}_{1}={\bar{\omega }}_{0}+\varepsilon \frac{\bar{\Gamma }N^{\left(0\right)}{\bar{\omega }}_{0}}{2A_{2}^{\left(0\right)}+A_{1}^{\left(0\right)}\beta (2-{\bar{\omega }}_{0}^{2})}+O(\varepsilon^{2})$$

Considering the relation (17) for $A_{1}^{\left(0\right)}$ and $A_{2}^{\left(0\right)}$, Equation (26) can be written as

(28) $$\bar{\omega }={\bar{\omega }}_{0}+\varepsilon \frac{3\bar{\Gamma }{\left|A_{2}^{\left(0\right)}\right|}^{2}{\bar{\omega }}_{0}^{7}}{2[1+\beta {\left(1-{\bar{\omega }}_{0}^{2}\right)}^{2}]}+O(\varepsilon^{2})$$

Substituting ${\bar{\omega }}_{0}^{opt}$ and ${\bar{\omega }}_{0}^{aco}$ into the above equation, the corresponding first-order asymptotic expansion of the dispersion curve equations for the acoustic and optical frequency branches can be obtained [18]:

(29) $${\bar{\omega }}_{opt}={\bar{\omega }}_{0}^{opt}+\varepsilon \frac{3\bar{\Gamma }{\left|A_{2}^{\left(0\right)}\right|}^{2}{\left({\bar{\omega }}_{0}^{opt}\right)}^{7}}{8[4+\beta {\left(2-{\left({\bar{\omega }}_{0}^{opt}\right)}^{2}\right)}^{2}]}$$

(30) $${\bar{\omega }}_{aco}={\bar{\omega }}_{0}^{aco}+\varepsilon \frac{3\bar{\Gamma }{\left|A_{2}^{\left(0\right)}\right|}^{2}{\left({\bar{\omega }}_{0}^{aco}\right)}^{7}}{8[4+\beta {\left(2-{\left({\bar{\omega }}_{0}^{opt}\right)}^{2}\right)}^{2}]}$$

These are the dispersion curve equations for the acoustic and optical frequency branches taking into account the nonlinearities, and it can be seen that the dispersion curve equations are related to the nonlinear perturbation let ε and the coefficients in front of the nonlinear terms $\bar{\Gamma }$ and the steady-state amplitude of the negatively stiffened Duffing oscillator $\left|A_{2}^{\left(0\right)}\right|$, and the mass ratio β.

**Figure 2:**
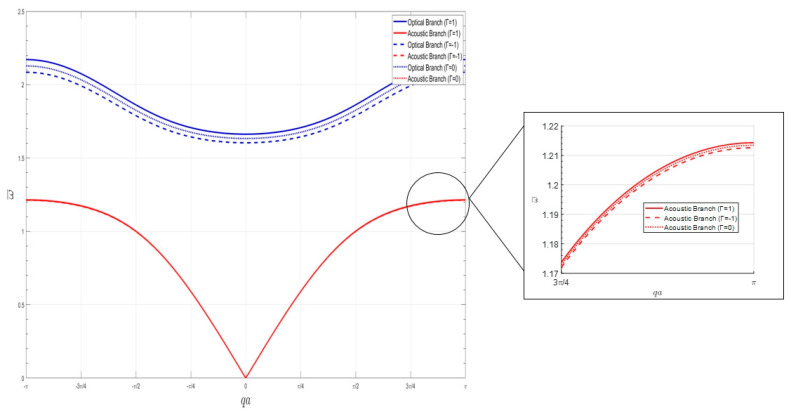
Effect of nonlinear factors on the dispersion curve.

Taking α=5,β=3,ε=0.0135, the effect of the coefficient Γ before the analytic nonlinear term on the dispersion curve is shown in Figure 2. Where Γ=1 is the hard-characteristic negative-stiffness Duffing oscillator, Γ=−1 is the soft-characteristic negative-stiffness Duffing oscillator, and Γ=0 is the linear system. As can be seen from the figure, when the local resonant oscillator is a hard-characteristic negative-stiffness Duffing oscillator, the two dispersion curves shift toward the high-frequency region relative to the linear system, resulting in an increase in both the bandgap starting frequency and cutoff frequency. Conversely, when the local resonant oscillator is a soft-characteristic negative-stiffness Duffing oscillator, the two dispersion curves shift toward the low-frequency region relative to the linear system, resulting in a decrease in both the bandgap starting frequency and cutoff frequency. Furthermore, under the same conditions, the nonlinear effect has a greater influence on the optical frequency branch dispersion equation than on the acoustic frequency branch dispersion equation. The stronger the nonlinearity, the more pronounced this trend becomes. In Figure 2, the blue curve represents the dispersion curve of the optical branch, while the red curve represents the dispersion curve of the acoustic branch. The solid lines correspond to Γ=1, the dashed lines correspond to Γ=−1, and the dotted lines correspond to Γ=0. As documented in Ref. [18], the bandgap initiation frequency for systems containing positive-stiffness Duffing oscillators is approximately 0.9 Hz, with a cutoff frequency of approximately 1.1 Hz. In contrast, Figure 2 of this study demonstrates that systems incorporating negative-stiffness Duffing oscillators exhibit a bandgap initiation frequency of approximately 1.2 Hz and a cutoff frequency of approximately 1.6 Hz. Compared to positive-stiffness systems, the bandgap initiation and cutoff frequencies in negative-stiffness systems shift to higher frequencies by a factor of $\sqrt{2}$, resulting in approximately $\sqrt{2}$ fold broadening of the bandgap width. From the perspective of bandwidth enhancement, these findings demonstrate a distinct advantage of negative-stiffness systems over their positive-stiffness counterparts.

### 2.4. Revisiting Starting and Cutoff Frequencies in Linear Systems

For the linear dispersion curve acoustic frequency branch ${\bar{\omega }}_{0}^{aco}$, the starting frequency of the bandgap is when qa=π

(31) $$\begin{array}{l} {\bar{\omega }}_{0}^{1}=\frac{\sqrt{\beta [\beta +1+\alpha -\sqrt{\alpha^{2}+2\alpha +1+\beta^{2}-2\beta -2\alpha \beta }]}}{\beta }\\ =\sqrt{\frac{2\beta +[1+\alpha -\beta -\sqrt{{\left(1+\alpha -\beta \right)}^{2}+4\beta }]}{\beta }}\\ =\sqrt{2-\frac{2}{1+\alpha -\beta +\sqrt{{\left(1+\alpha -\beta \right)}^{2}+4\beta }}} \end{array}$$

**Figure 3:**
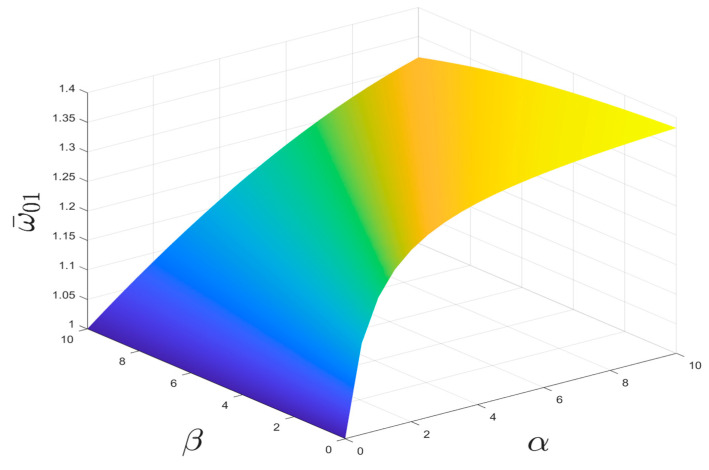
Relationship of α, β, and starting frequency ${\bar{\omega }}_{01}$.

It is easy to prove that $1+\alpha -\beta +\sqrt{{\left(1+\alpha -\beta \right)}^{2}+4\beta }>0$, so that the starting frequency of the dimensionless bandgap is less than $\sqrt{2}$. When α and β take different values, the variation curves of the starting frequency are shown below in Figure 3.

As can be seen in Figure 3, the law of change in the starting frequency ${\bar{\omega }}_{01}$ after dimensionless normalization is not only related to the ratio of the negative-stiffness Duffing oscillator to the linear stiffness of the shell α, but also to the ratio of the mass of the negative-stiffness Duffing oscillator to the shell β.

**Figure 4:**
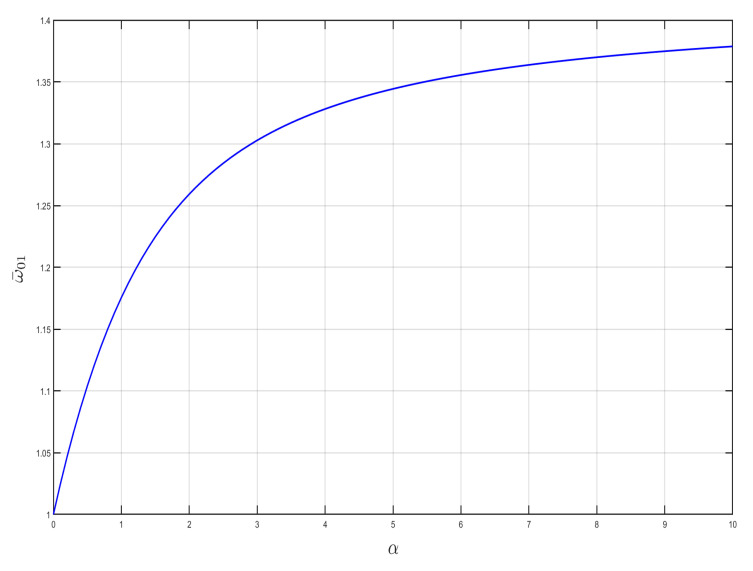
Effect of α on starting frequency ${\bar{\omega }}_{01}$(β=1).

**Figure 5:**
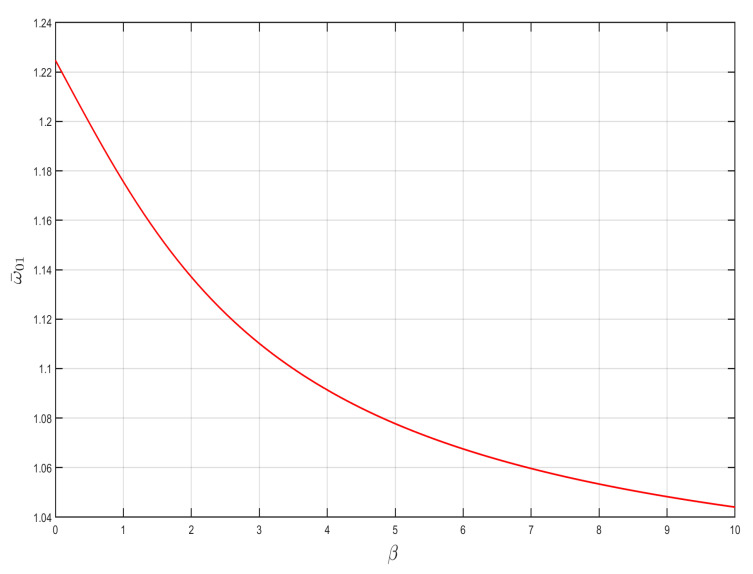
Effect of β on starting frequency ${\bar{\omega }}_{01}$(α=1).

The effect of α and β on the starting frequency ${\bar{\omega }}_{01}$ is investigated by taking the stiffness ratio and mass ratio of the constant oscillator and shell, respectively, as shown in Figure 4 and Figure 5. It can be seen from the figures that the starting frequency ${\bar{\omega }}_{01}$ monotonically increases with stiffness ratio α and monotonically decreases with linear mass ratio β for the same case. The larger the linear stiffness ratio, the higher the starting frequency, and the smaller the stiffness ratio, the lower the starting frequency.

For a linear dispersion curve optical frequency branch ${\bar{\omega }}_{0}^{opt}$, the cutoff frequency of the bandgap is when qa=0:

(32) $${\bar{\omega }}_{02}=\sqrt{2(1+\frac{1}{\beta })}$$

Equation (32) shows that the bandgap cutoff frequency is only related to the oscillator mass ratio and becomes lower as the mass ratio increases.

By comparison with the positive-stiffness linear system, it can be seen that the starting frequency of the negative-stiffness linear system is slightly less than $\sqrt{2}$ times the starting frequency of the corresponding positive-stiffness linear system, while its cutoff frequency is exactly $\sqrt{2}$ times the cutoff frequency of the corresponding positive-stiffness linear system.

To demonstrate the superiority of the negative-stiffness vibration reduction system, the results obtained in this work are compared with those reported in Ref. [18], as summarized in Table 1. As illustrated in the table, the negative-stiffness system exhibits a broader bandgap width compared to its positive-stiffness counterpart. This finding provides strong technical evidence supporting the development of vibration reduction systems with ultra-broadband bandgap performance and further validates the comparative advantage of negative-stiffness systems over positive-stiffness systems.

## 3. Numerical Verification

**Figure 6:**
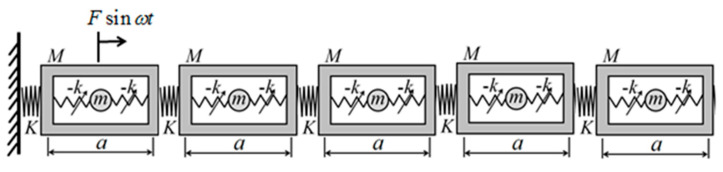
Five periodic negative effective mass system containing negative-stiffness Duffing oscillator.

In order to verify the correctness of the above theoretical analysis, the five-period effective negative mass system containing a negative-stiffness Duffing vibrator is now modeled as shown in Figure 6. A simple harmonic excitation force of Fsin(ωt) is added to the shell in the first period, and the shell picks up the response in the fifth period, corresponding to each excitation frequency, and the steady-state displacements of the shell and the negative-stiffness Duffing oscillator are calculated for each period, so as to obtain the vibration-transfer characteristics of the finite-period structure, and analyze the vibration-transfer law of the structure in order to obtain the bandgap range.

The forces are analyzed for the shell and the negative stiffness Duffing oscillator for each period, respectively, and a system of differential equations of motion for the system is established according to Newton’s second law, as follows:

(33) $$\left\{\begin{array}{l} M{\ddot{x}}_{1}+K(2x_{1}-x_{3})+c(2{\dot{x}}_{1}-{\dot{x}}_{3})+2k(x_{2}-x_{1})-2\varepsilon \Gamma {\left(x_{2}-x_{1}\right)}^{3}=F\sin(\omega t)\\ m{\ddot{x}}_{2}-2k(x_{2}-x_{1})+2\varepsilon \Gamma {\left(x_{2}-x_{1}\right)}^{3}=0\\ M{\ddot{x}}_{3}+K(2x_{3}-x_{1}-x_{5})+c(2{\dot{x}}_{3}-{\dot{x}}_{1}-{\dot{x}}_{5})+2k(x_{4}-x_{3})-2\varepsilon \Gamma {\left(x_{4}-x_{3}\right)}^{3}=0\\ m{\ddot{x}}_{4}-2k(x_{4}-x_{3})+2\varepsilon \Gamma {\left(x_{4}-x_{3}\right)}^{3}=0\\ M{\ddot{x}}_{5}+K(2x_{5}-x_{3}-x_{7})+c(2{\dot{x}}_{5}-{\dot{x}}_{3}-{\dot{x}}_{7})+2k(x_{6}-x_{5})-2\varepsilon \Gamma {\left(x_{6}-x_{5}\right)}^{3}=0\\ m{\ddot{x}}_{6}-2k(x_{6}-x_{5})+2\varepsilon \Gamma {\left(x_{6}-x_{5}\right)}^{3}=0\\ M{\ddot{x}}_{7}+K(2x_{7}-x_{5}-x_{9})+c(2{\dot{x}}_{7}-{\dot{x}}_{5}-{\dot{x}}_{9})+2k(x_{8}-x_{7})-2\varepsilon \Gamma {\left(x_{8}-x_{7}\right)}^{3}=0\\ m{\ddot{x}}_{8}-2k(x_{8}-x_{7})+2\varepsilon \Gamma {\left(x_{8}-x_{7}\right)}^{3}=0\\ M{\ddot{x}}_{9}+K(x_{9}-x_{7})+c({\dot{x}}_{9}-{\dot{x}}_{7})+2k(x_{10}-x_{9})-2\varepsilon \Gamma {\left(x_{10}-x_{9}\right)}^{3}=0\\ m{\ddot{x}}_{10}-2k(x_{10}-x_{9})+2\varepsilon \Gamma {\left(x_{10}-x_{9}\right)}^{3}=0 \end{array}\right.$$

where $x_{1},x_{3},x_{5},x_{7,}x_{9}$ represents the displacements of the one- to five-period shells, respectively, while $x_{2},x_{4},x_{6},x_{8,}x_{10}$ represents the displacements of the one- to five-period negative-stiffness Duffing oscillators, respectively. If the nonlinear perturbation term ε=0.01 is taken, it is defined for ease of presentation:

(34) Γ=nk

when *n* is positive, the negative-stiffness Duffing oscillator is asymptotically stiff and hard. In contrast, when *n* is negative, the negative-stiffness Duffing oscillator is stiff asymptotically soft, and when *n* is zero, the system is a degenerate linear system.

### 3.1. Widening of the Bandgap Frequency Range

**Figure 7:**
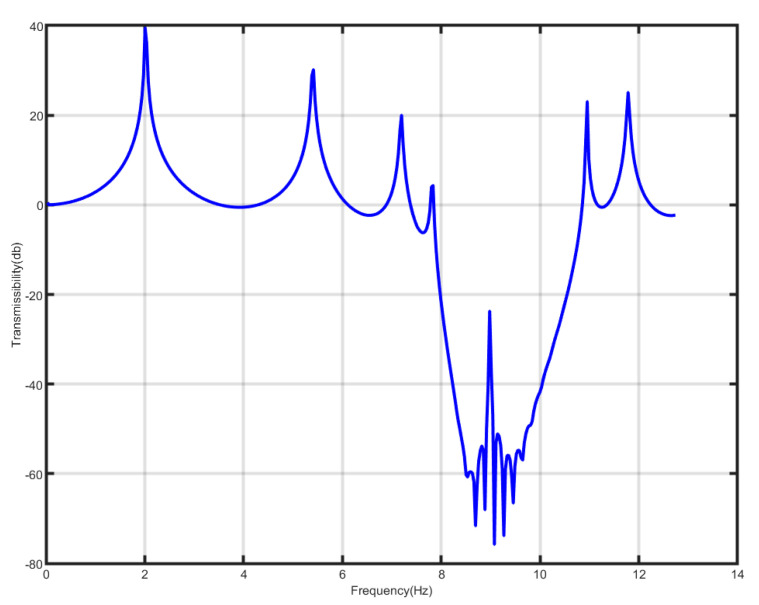
Bandgap of metamaterial containing negative-stiffness Duffing oscillator.

To validate the aforementioned theoretical findings, parameters identical to those in Ref. [18] were selected, with the exception that the linear stiffness of the internal nonlinear spring was set to negative values, the mass of the outer shell *M* = 0.1011 kg, the mass of the internal negative-stiffness Duffing vibrator *m* = 0.04647 kg, the linear stiffness of the internal nonlinear spring *K* = −37 N/m, the damping coefficient of the external linear spring *c* = 0.05 NS/m, and the stiffness of the spring connected with the external large vibrator coefficient *K* = 117 N/m, if we take the nonlinear perturbation term ε=0.01 and calculate the vibration transmittance of this periodic structure, as shown in Figure 7, we can get its bandgap range between 7.9 and 10.8 Hz. The bandgap for the positive-stiffness Duffing oscillators documented in Ref. [18] ranges from 5.67 to 7.67 Hz. The starting frequency of the negative Duffing oscillator system is slightly smaller than $\sqrt{2}$ times of the starting frequency of the corresponding positive-stiffness system, while its cutoff frequency is very close to $\sqrt{2}$ times of the cutoff frequency of the positive-stiffness system, which makes the whole bandgap of the negative-stiffness system shifted to the high frequency and the width of the negative-stiffness system become nearly $\sqrt{2}$ times of the positive-stiffness system, and the width of the bandgap becomes bigger, so that the system with negative stiffness is better than the system with positive-stiffness Duffing oscillator. From this point of view, the system with negative-stiffness Duffing oscillator is better than the system with positive-stiffness Duffing oscillator, consistent with the theoretical analysis.

### 3.2. Effect of Shell Stiffness on Bandgap Frequency

**Figure 8:**
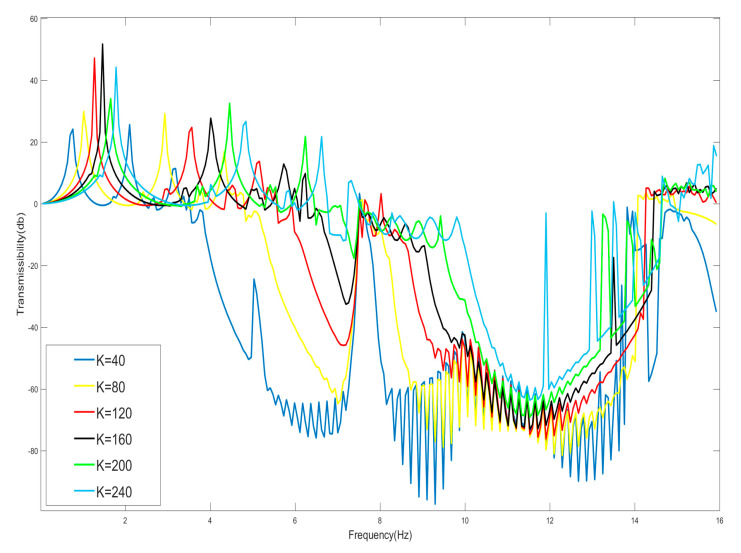
Effect of shell oscillator stiffness *K* on starting frequency.

Due to the presence of weakly nonlinear terms, there are small variations in the starting and cutoff frequencies of the bandgap for the corresponding linear system, with the starting frequency increasing monotonically with the stiffness ratio α and decreasing monotonically with the mass ratio β, whereas the cutoff frequency decreases monotonically only with the mass ratio β. The value of stiffness factor *K* of the spring between the shells is now varied to discuss the effect of stiffness ratio α on the bandgap generation. Here, m=0.125 kg, M=0.1 kg, k=−123.2 N/m, *n* = 1, *K* = 40, 80, 120, 160, 200, 240 N/m, are taken and the vibration-transfer characteristics of each system are plotted as shown in Figure 8.

Since the selected negative-stiffness Duffing oscillator is weakly nonlinear, the presence of the nonlinearity has a limited effect on the bandgap, which is only slightly changed in the neighborhood of the linear system after its degradation. And when the shell connection stiffness *K* is gradually made smaller, in the case of negative-stiffness Duffing oscillator and the mass of the shell remaining unchanged, according to the analytical results of Equations (31) and (32) and Figure 4 and Figure 5, the cutoff frequency of the bandgap does not change after reducing the shell stiffness, but the starting frequency decreases, the bandwidth of the bandgap is increased, and the bandgap expands to the low-frequency band. From Figure 8, it can be seen that the cutoff frequency of the bandgap is 15 Hz, which does not change, and the starting frequency of the bandgap becomes lower due to the reduction in the enclosure stiffness. Therefore, to obtain a large bandgap width, the stiffness coefficient of the linear spring connected to the housing should be as small as possible, all other conditions being equal.

Moreover, the linear resonance frequency of the internal oscillator is $\omega_{0}=\sqrt{2}\frac{1}{2\pi }\sqrt{\frac{2k}{m}}=\sqrt{2}\times \frac{1}{2\pi }\sqrt{\frac{2\times 123.2}{0.125}}=10$ Hz, and this frequency point corresponds to the frequency value of the beginning of the effective negative mass; the analysis according to Equation (32) shows that 15 Hz is the frequency value of the zero mass, and therefore the 10 Hz to 15 Hz corresponds to the effective negative mass region. And the frequency below 10 Hz in the bandgap range should correspond to the effective positive mass region.

### 3.3. Effect of Excitation Force Amplitude on the Starting and Cutoff Frequencies of the Bandgap

**Figure 9:**
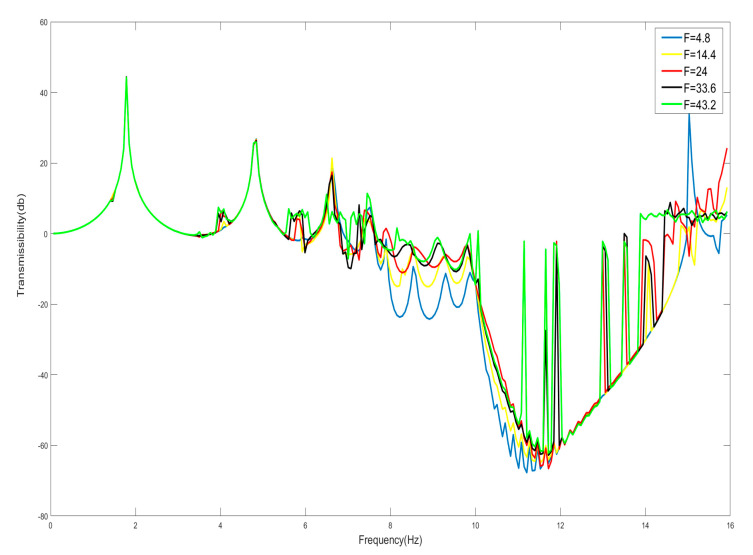
Effect of external excitation amplitude *F* on bandgap.

For nonlinear systems, the response of the system tends to be sensitive to the amplitude of the external excitation. The response characteristics of the nonlinear system may change with the change in the amplitude of the external excitation. For this reason, the effect of the change in the amplitude of the external excitation on the bandgap is analyzed here. And here we take the case of m=0.125 kg, M=0.1 kg, k=−123.2 N/m, *n* = 1, *K* = 240 N/m, and the smaller amplitude, according to Figure 9, shows that its bandgap should be in the range of 7.53–15 Hz, and the increase in the amplitude of the external excitation, amplitude *F* are 4.8 N, 14.4 N, 24 N, 33.6 N, and 43.2 N, respectively. And observe the change in the bandgap width as shown in Figure 9. From Figure 9, it can be seen that when the amplitude gradually increases, the starting frequency of the bandgap gradually increases, while the cutoff frequency decreases, resulting in a decrease in the bandwidth of the entire bandgap; for example, when the amplitude of the excitation force is less than 33.6 N, 14–15 Hz was originally within the bandgap, but when the amplitude is increased to 43.2 N, the range of 14–15 Hz becomes the passband, which will have an adverse effect on the bandgap design disadvantage.

**Figure 10:**
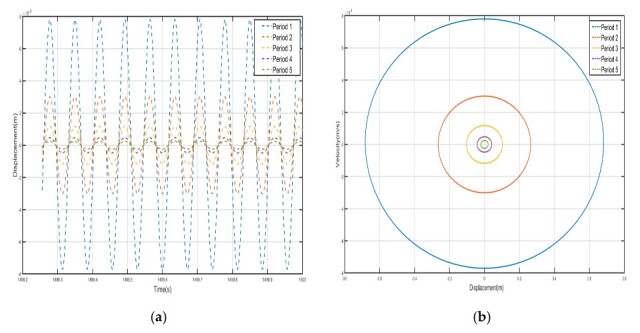
Five periodic shells’ (**a**) displacement–time history curves and (**b**) velocity-displacement phase diagrams. (*F* = 4.8 N, 14 Hz).

**Figure 11:**
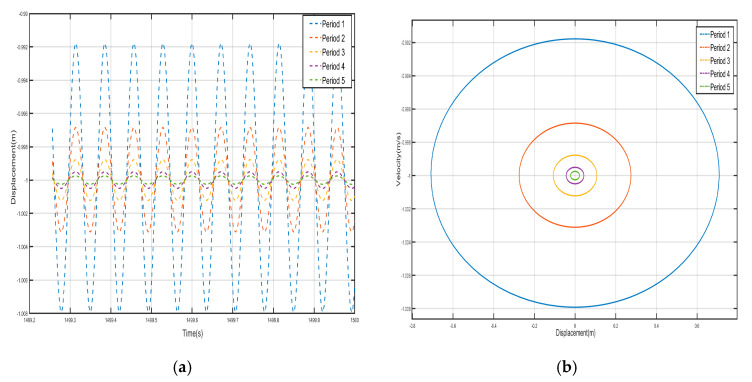
Five periodic negative-stiffness Duffing oscillators’ (**a**) displacement–time history curves and (**b**) velocity-displacement phase diagrams. (*F* = 4.8 N, 14 Hz).

Take *F* = 4.8 N, the external excitation frequency is 14 Hz, draw five periods of shell and internal negative-stiffness Duffing vibration displacement–time history curves and velocity-displacement phase diagrams shown in Figure 10 and Figure 11.

From the curves of the vibration displacement versus time of each shell and negative-stiffness Duffing oscillator in Figure 10 and Figure 11, it can be observed that after a certain period, the amplitude of the vibration of each shell and oscillator no longer decays and reaches a stable value. Moreover, the velocity-displacement phase diagrams of the shells and oscillators are all ellipses, indicating that each shell and oscillator of this periodic structure has reached a steady-state vibration.

From Equations (6) and (34), it can be seen that the shell and the negative-stiffness Duffing vibrator should vibrate near their respective stable equilibrium positions after reaching the steady state, and the stable equilibrium points of the shell and the negative-stiffness Duffing vibrator are, respectively, when *n* = 1

(35) $$\left\{\begin{array}{c} v_{1}=0\\ v_{2}=\pm 1 \end{array}\right.$$

where $v_{1}$ is the stabilized equilibrium point position of the shell and $v_{2}$ is the stabilized equilibrium point position of the negative-stiffness Duffing vibrator.

Comparing the shell vibration of each period in Figure 10 and Figure 11, from the first period to the fifth period, the shell vibrates near its stable equilibrium point, and comparing the displacement amplitude of each shell and oscillator, it can be found that the amplitude has a tendency to decay. The first period of the shell displacement amplitude decayed by about $8\times 10^{-3}$ m, while the second period of the shell displacement amplitude decayed by only $3\times 10^{-3}$ m, to the third period of the attenuation by $1.12\times 10^{-3}$ m, the fourth period of the further attenuation by $5\times 10^{-4}$ m, and to the fifth period of only about $2.5\times 10^{-4}$ m; the vibration displacements are very small and a bandgap appears at a frequency value corresponding to the external excitation frequency of 14 Hz. Similarly, the vibration of the internal negative-stiffness Duffing vibrator is very similar to the case of the shell, and each negative-stiffness Duffing vibrator vibrates at its respective stable equilibrium position $v_{2}=-1$ with the vibration of each period also decreasing gradually; the amplitude of the displacement of the negative-stiffness Duffing vibrator from the first to the fifth period is $7\times 10^{-3}$, $3.2\times 10^{-3}$, $1.2\times 10^{-3}$, $5\times 10^{-4}$, and $3\times 10^{-4}$, in that order, and in the first period of the negative-stiffness Duffing vibrator, the vibration of the oscillator cannot be transmitted and is suppressed; this phenomenon is in line with the characteristics of the bandgap.

**Figure 12:**
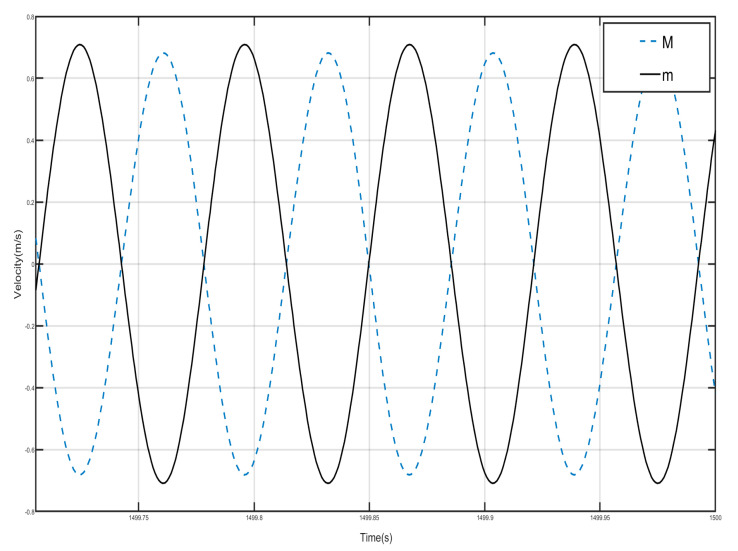
First periodic shell’s and negative-stiffness Duffing oscillator’s vibration velocity–time history curves. (*F* = 4.8 N, 14 Hz).

Draw the first period of the shell and Duffing vibration velocity–time history curves, as shown in Figure 12. Figure 12 has a dashed line for the velocity–time history curve of the shell, and a solid line for the negative-stiffness Duffing vibrator velocity–time history curve; from the figure, it can be seen that when the shell M motion speed reaches the negative amplitude, the negative-stiffness Duffing vibrator m has movement to the positive amplitude, and when the M motion speed has the positive amplitude, the m motion speed has the negative amplitude, so the M and m of the motion speed is always the opposite of shell and vibration at anti-phase. Since the selected m mass is larger than M, under the same external excitation amplitude, the object with smaller mass is more likely to change its velocity, so the corresponding displacement is larger. Further analysis can be obtained; when the momentum of the internal negative-stiffness Duffing vibrator is greater than the momentum of the shell, then a single-period shell and negative stiffness Duffing vibrator composed of the system has a negative momentum, because the speed is positive so the system has a negative mass, and the system plays the role of vibration-damping vibration absorption.

**Figure 13:**
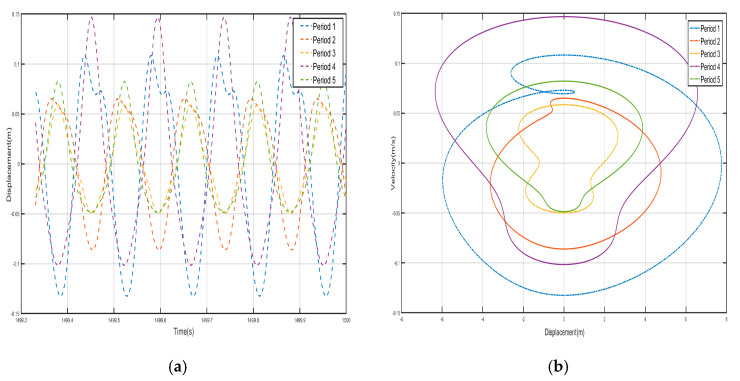
Five periodic shells’ (**a**) displacement–time history curves and (**b**) velocity-displacement phase diagrams. (*F* = 24 N, 14 Hz).

**Figure 14:**
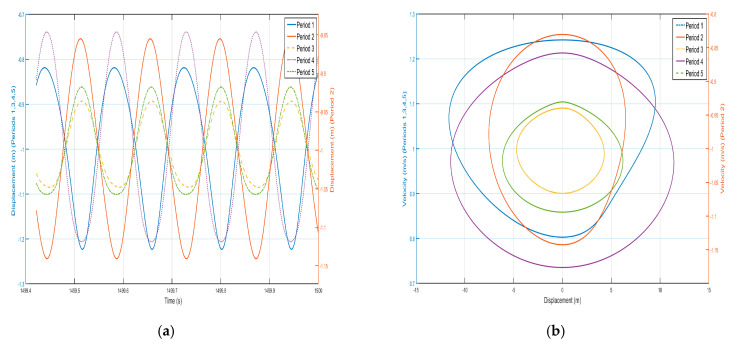
Five periodic negative-stiffness Duffing oscillators’ (**a**) displacement–time history curves and (**b**) velocity-displacement phase diagrams. (*F* = 24 N, 14 Hz).

Take *F =* 24 N, the external excitation frequency is still 14 Hz, five periods of shell and internal negative-stiffness Duffing vibration displacement–time history curves and velocity-displacement phase diagrams shown in Figure 13 and Figure 14. From Figure 13 and Figure 14, each shell and negative-stiffness Duffing vibration displacement–time history curve, after a certain period of time, show that each shell and vibration amplitude of vibration is no longer at attenuation; to reach a stable value, the period structure of each shell and negative-stiffness Duffing vibration must have reached a steady-state vibration. Moreover, due to the large amplitude of vibration, the displacement of each shell and negative-stiffness Duffing vibrator have relatively increased greatly.

According to the vibration of the shell in each period of Figure 13 and Figure 14, from the first period to the fifth period, although the shell still vibrates near its stable equilibrium point, the vibration of each period does not show a trend of gradual decrease. For the vibration of the first to the fifth period, we analyze the displacement amplitude of the shell and the oscillator. The displacement amplitudes of the shell from the first period to the fifth period are $1.08\times 10^{-1}$ m, $7.5\times 10^{-2}$ m, $6\times 10^{-2}$ m, $1.5\times 10^{-1}$ m, and $9\times 10^{-2}$ m, respectively. The displacement amplitude not only does not show a trend of attenuation, but also enlarges in the fourth period, indicating that the vibration can be transmitted to the following period, which is not a typical bandgap. Therefore, there is no bandgap when the corresponding external excitation frequency is 14 Hz. Similarly, the vibration characteristics of the internal negative-stiffness Duffing oscillator are very similar to those of the shell. The negative-stiffness Duffing oscillator in the second period vibrates near its stable equilibrium position $v_{2}=\;-1$, and the negative-stiffness Duffing oscillator in the other periods vibrates near its stable equilibrium position $v_{2}=\;1$. The displacement amplitudes of the negative-stiffness Duffing oscillator in the first to fifth periods are 0.21 m, 0.15 m, 0.1 m, 0.25 m, and 0.15 m, respectively. Similarly, the displacement amplitude of the negative-stiffness Duffing oscillator does not show an attenuation trend, but it is amplified in the fourth period. It shows that the propagation of vibration is not suppressed, and it is not a typical bandgap characteristic.

**Figure 15:**
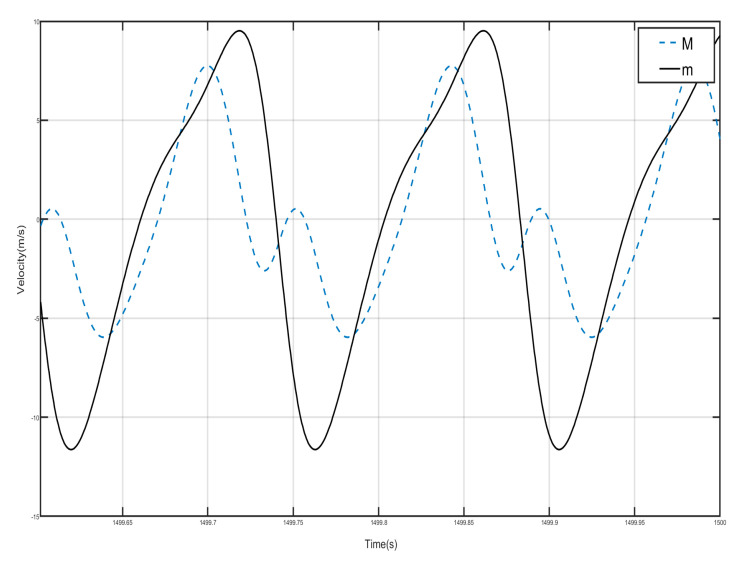
First periodic shell’s and negative-stiffness Duffing oscillator’s vibration velocity–time history curves. (*F* = 24 N, 14 Hz).

Draw the velocity–time history curves of the shell and the negative-stiffness Duffing vibrator during the first period of vibration, as shown in Figure 15. The dashed line in Figure 15 is the velocity–time history curve of the shell, and the solid line is the velocity–time history curve of the negative-stiffness Duffing vibrator. It can be seen from the figure that when the motion velocity of the shell *M* reaches the positive amplitude, the motion velocity of the negative-stiffness Duffing vibrator *m* does not reach the negative amplitude, and when the motion velocity of *M* is the negative amplitude, the motion velocity of *m* does not reach the positive amplitude, so the motion velocity of *M* and *m* are not always opposite; most of the time the motion of the shell and the internal negative-stiffness Duffing vibrator is not always opposite, and most of the time the motions of the shell and the internal negative-stiffness Duffing oscillator are in the same direction. Further analysis can be obtained that the momentum of the internal negative-stiffness Duffing oscillator and the shell add up to a positive value, and the cell is of effective positive mass.

The above analysis shows that, for the effective negative mass period structure containing negative-stiffness Duffing vibrator, when the amplitude of the external excitation becomes large, it will lead to the narrowing of the bandgap range, and when the amplitude of the external excitation is small, the frequency range originally located in the bandgap, when the external excitation is large, does not have obvious characteristics of the bandgap, and it may even be changed into the frequency range of the passband. Therefore, when using a negative-stiffness Duffing vibrator containing an effective negative mass period structure for vibration isolation, the amplitude of the external excitation for the need of vibration and noise reduction cannot be too large.

### 3.4. Vibrational Response of Shell and Oscillators Within the Bandgap Frequency Range

It has been known from the previous discussion of the bandgap starting frequency that, similarly to periodic structures containing positively stiffened Duffing oscillators, the presence of weakly nonlinear factors only varies marginally around the value of the frequency of their degenerate linear system, but the negatively stiffened Duffing oscillators and shells with linear stiffness ratios of α and mass ratios of β both produce variations in the starting frequency. Furthermore, the starting frequency is lower than that of the linear oscillator’s resonant frequency of the linear oscillator. That is, the bandgap is also not always due to the effective negative mass. In the bandgap range, the negatively stiffened Duffing oscillator and the shell should have effective positive mass below the linear resonance frequency for each period, and effective negative mass below the cutoff frequency above the resonance frequency. For effective comparison, m=0.125 kg, *M* = 0.1 kg, k=−123.2 N/m, *n* = 1, *K* = 240 N/m are still taken here, and the vibration-transfer characteristics of their corresponding systems are shown in Figure 9. Moreover, the frequency at 10 Hz in the bandgap range corresponds to the frequency value of the start of the effective negative mass, and 15 Hz is the frequency value of the zero mass, so 10 Hz to 15 Hz corresponds to the effective negative mass region. And the frequencies below 10 Hz in the bandgap range should correspond to the effective positive mass region.

In the previous analysis it is known that when *F* = 4.8 N and the external excitation frequency is 14 Hz, the cell corresponds to an effective negative mass when located at 14 Hz in the 10–15 Hz bandgap range as shown in Figure 10, Figure 11 and Figure 12.

**Figure 16:**
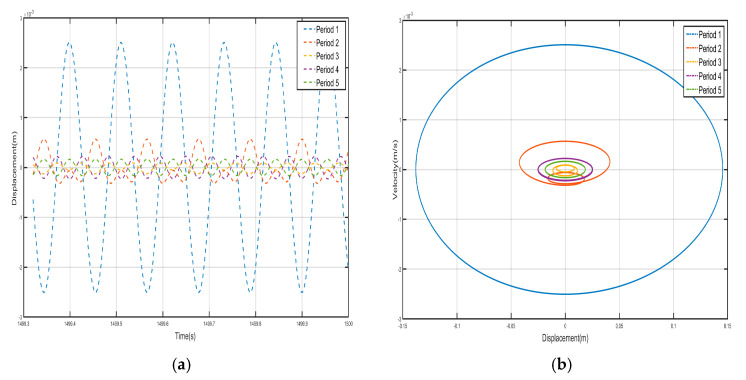
Five periodic shells’ (**a**) displacement–time history curves and (**b**) velocity-displacement phase diagrams. (*F* = 4.8 N, 9 Hz).

**Figure 17:**
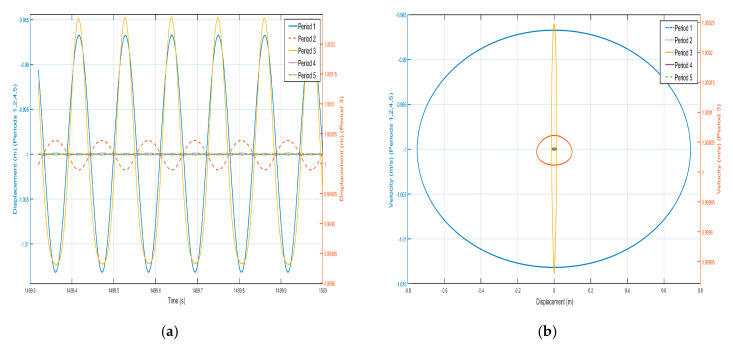
Five periodic negative-stiffness Duffing oscillators’ (**a**) displacement–time history curves and (**b**) velocity-displacement phase diagrams. (*F* = 4.8 N, 9 Hz).

We take *F* = 4.8 N and the external excitation frequency is 9 Hz to analyze the response of each shell and vibrator of the system in the effective positive mass region within the bandgap range, and draw the vibration displacement–time history curves and velocity-displacement phase diagrams of the five-period shells and the internal negative-stiffness Duffing vibrator as shown in Figure 16 and Figure 17.

Comparing the shell vibration in each period of Figure 16 and Figure 17, starting from the first period to the fifth period, the shell vibrates near its stable equilibrium point, and the amplitude of the shell displacement is about $2.2\times 10^{-2}$ m in the first period, while the amplitude of the shell displacement is only $1.5\times 10^{-2}$ m in the second period, and it attenuates to $8\times 10^{-3}$ m in the third period, and then attenuates to $1.3\times 10^{-3}$ m in the fourth period, while it is about m in the $6\times 10^{-3}$ fifth period. The vibration displacement decreases from the first period to the fourth period, and although it increases slightly in the fifth period, the vibration displacement decreases by more than three times with respect to the vibration case in the first period, indicating that the propagation of vibration is suppressed as a whole. Similarly, the vibration characteristics of the internal negative-stiffness Duffing vibrators are very similar to the case of the shell, with each negative-stiffness Duffing vibrator vibrating at its stable equilibrium position, with the negative-stiffness Duffing vibrators vibrating at $v_{2}=-1$ for the first, second, fourth, and fifth periods, and the negative-stiffness Duffing vibrators vibrating at $v_{2}=1$ for the third periods. The displacement amplitudes of the negative-stiffness Duffing vibrators from the first to the fifth period are $5\times 10^{-2}$ m, $2\times 10^{-2}$ m, $1.35\times 10^{-2}$ m, $4.8\times 10^{-3}$ m, and $5\times 10^{-3}$ m in that order. The vibration displacements of the enclosure are decreasing from the first period to the fourth period, and although they slightly increase in the fifth period, the vibration displacements decrease by nearly 10 times with respect to the vibration situation in the first period, indicating that the propagation of vibration is suppressed overall and belongs to the range of the bandgap.

**Figure 18:**
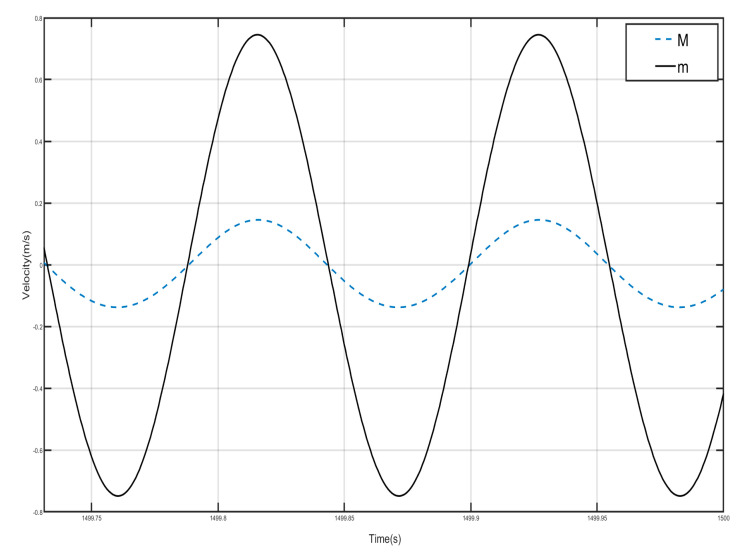
First periodic shell’s and negative-stiffness Duffing oscillator’s vibration velocity–time history curves. (*F* = 4.8 N, 9 Hz).

Draw the velocity–time history curves of the shell and the Duffing vibrator when they vibrate in the first period, as shown in Figure 18. The dotted line in Figure 18 is the velocity–time history curve of the shell, and the dashed line is the velocity–time history curve of the negative-stiffness Duffing vibrator, from which it can be seen that when the velocity of the shell *M* motion reaches a positive value, the velocity of the negative-stiffness Duffing vibrator *m* motion is also positive, and when the velocity of the *M* motion is negative, the velocity of the *m* motion is also positive, i.e., the velocity of the *M* and the *m* motion are in the same direction, and then the system consisting of a single periodic shell and a negative-stiffness Duffing vibrator has positive momentum, because the velocity is positive, so the system has an effective positive mass, and the system plays the role of vibration absorption and damping, but is still in the bandgap range.

### 3.5. Response of Shells and Oscillators in the Passband Section

**Figure 19:**
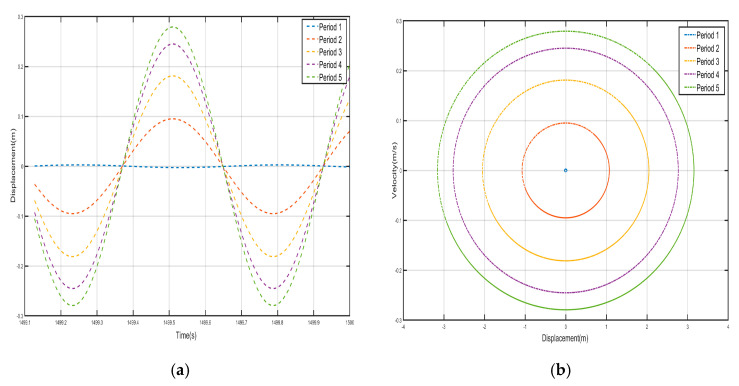
Five periodic shells’ (**a**) displacement–time history curves and (**b**) velocity-displacement phase diagrams. (*F* = 24 N, 1.8 Hz).

**Figure 20:**
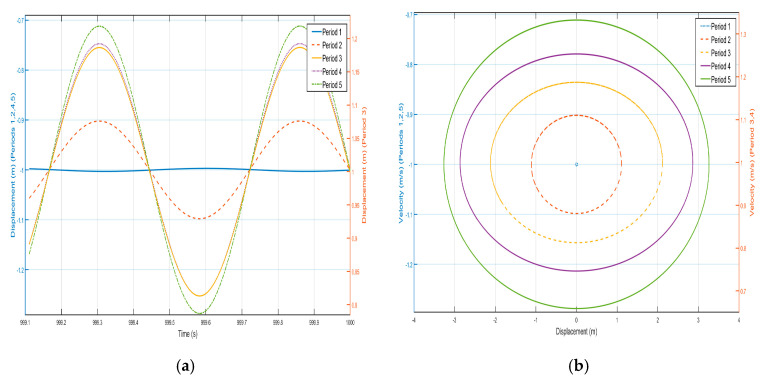
Five periodic negative-stiffness Duffing oscillators’ (**a**) displacement–time history curves and (**b**) velocity-displacement phase diagrams. (*F* = 24 N, 1.8 Hz).

The previous section analyses the characteristics of the bandgap range, which contains both effective negative and positive masses. The shell and oscillator responses in the passband section are analyzed below. The external excitation frequency of ω=1.8 Hz is selected, and the displacement–time history curves and velocity-displacement phase diagrams here are analyzed, as shown in Figure 19 and Figure 20. From the displacement–time history curves of each shell and negative-stiffness Duffing oscillator shown in the figure, it can be seen that after a certain period of time, the amplitude of each shell and oscillator vibration no longer decays and reaches a stable value. Furthermore, the velocity-displacement phase diagrams of the shells and oscillators are both elliptical, indicating that the vibrations of each shell and oscillator in this periodic structure have reached a steady state.

In Figure 19 and Figure 20, against the shell vibration of each period, from the first period to the fifth period, the vibration of the shell not only does not show a tendency of attenuation but also shows a tendency of increasing step by step. For example, the displacement amplitudes of the shell from the first period to the fifth period are $1.15\times 10^{-2}$ m, $8\times 10^{-2}$ m, 0.15 m, 0.218 m, and 0.25 m, respectively, and the vibration in the first period can be well transmitted to the fifth period. Similarly, the oscillation of the internal negative-stiffness Duffing oscillator exhibits analogous behavior to the outer shell. Specifically, as observed in the displacement–time history curves, the negative-stiffness Duffing oscillator during the third period oscillates about its stable equilibrium point at v= 1, whereas during the first, second, fourth, and fifth periods, it oscillates about its stable equilibrium point at v= −1. Additionally, analysis of the phase portraits reveals that the negative-stiffness Duffing oscillator during the third and fourth periods oscillates about its stable equilibrium point at v= 1, while during the first, second, and fifth periods, it oscillates about its stable equilibrium point at v= −1, and the amplitudes of the negative-stiffness Duffing vibration of the first period to the fifth period are $1.12\times 10^{-2}$ m, $8.1\times 10^{-2}$ m, 0.16 m, 0.215 m, 0.258 m, respectively. The amplitude of each negative-stiffness Duffing vibration also shows a tendency to amplify step by step, which indicates that the vibration loaded on the shell in the first period is well propagated in the structure of the period, and there is no inhibition, so that there is no bandgap at the frequency, which is in line with the characteristics of the passband.

**Figure 21:**
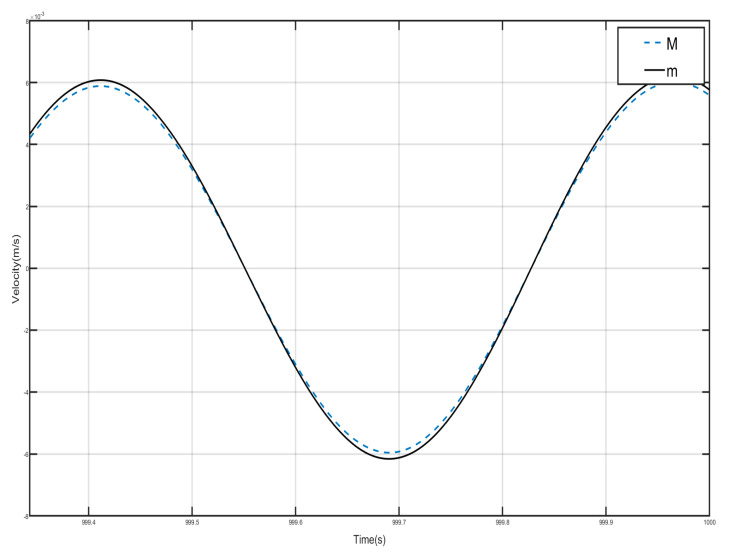
First periodic shell’s and negative-stiffness Duffing oscillator’s vibration velocity–time history curves. (*F* = 24 N, 1.8 Hz).

The velocity–time history curves of the shell and Duffing vibrator after steady state of the first period are drawn, as shown in Figure 21; the dotted line is the velocity–time history curve of the shell, and the solid line is the velocity–time history curve of the negative-stiffness Duffing vibrator. It can be seen that when the shell *M* motion speed reached the positive amplitude, negative-stiffness Duffing vibrator *m* motion speed also reached the positive amplitude, when the shell *M* motion speed reached the negative amplitude, *m* motion speed is also negative amplitude, so the *M* and *m* motion speed is always the same direction, and the velocity values of the shell and the negative-stiffness Duffing oscillator are very close, so the periodic unit conforms to the characteristics of effective positive mass.

The change trend of the velocity–time history curves of the shell and the negative-stiffness Duffing oscillator from the second to the fourth period is similar to that of Figure 21, both of which show the characteristics of effective positive mass.

### 3.6. Discussion of Zero Mass

**Figure 22:**
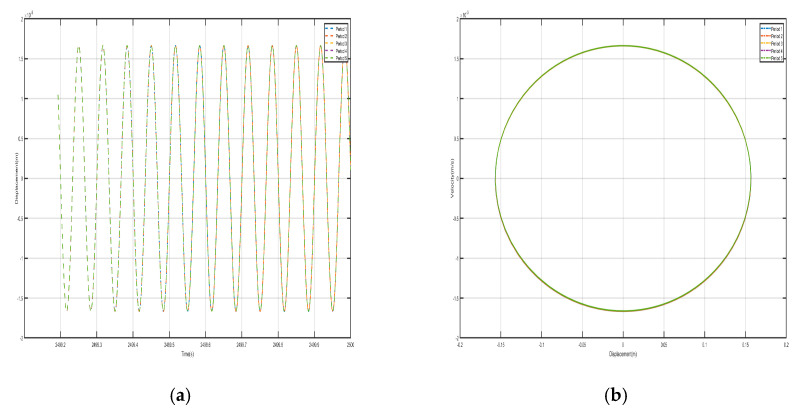
Five periodic shells’ (**a**) displacement–time history curves and (**b**) velocity-displacement phase diagrams. (zero mass).

**Figure 23:**
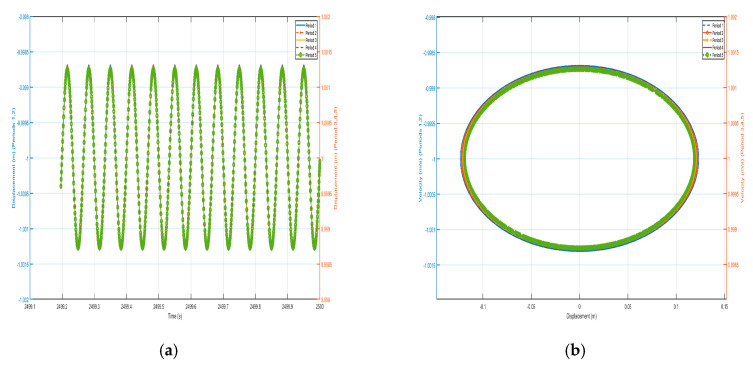
Five periodic negative-stiffness Duffing oscillators’ (**a**) displacement–time history curves and (**b**) velocity-displacement phase diagrams. (zero mass).

According to the results of the previous analysis, the frequency at zero-mass corresponds to the cutoff frequency of the bandgap. The presence of nonlinear factors affects the frequency at zero mass; Accordingly, by selecting frequencies with closely spaced values, the bandgap cutoff frequency of the nonlinear system is derived, which corresponds to the frequency at zero mass. We find the frequency point corresponding to zero mass to be 14.993 Hz, and draw the five periodic shells’ and negative-stiffness Duffing oscillators’ displacement–time history curves and velocity-displacement phase diagrams., as shown in Figure 22 and Figure 23. From the figure, we can see that the shell and the internal negative-stiffness Duffing vibrator of each period of the system are in motion, and from the velocity-displacement phase diagrams, we can see that the shell and the Duffing vibrator have reached the steady state, and the maximum displacements of the shell and the Duffing vibrator of each period are the same. From the displacement–time history curves, it is observed that the negative-stiffness Duffing oscillator during the second period oscillates about its stable equilibrium point at v= 1, whereas during the first, third, fourth, and fifth periods, it oscillates about its stable equilibrium point at v= −1. Analysis of velocity-displacement phase diagrams., however, reveals that the negative-stiffness Duffing oscillator during the third, fourth, and fifth periods oscillates about its stable equilibrium point at v= 1, while during the first and second periods, it oscillates about its stable equilibrium point at v= −1.

**Figure 24:**
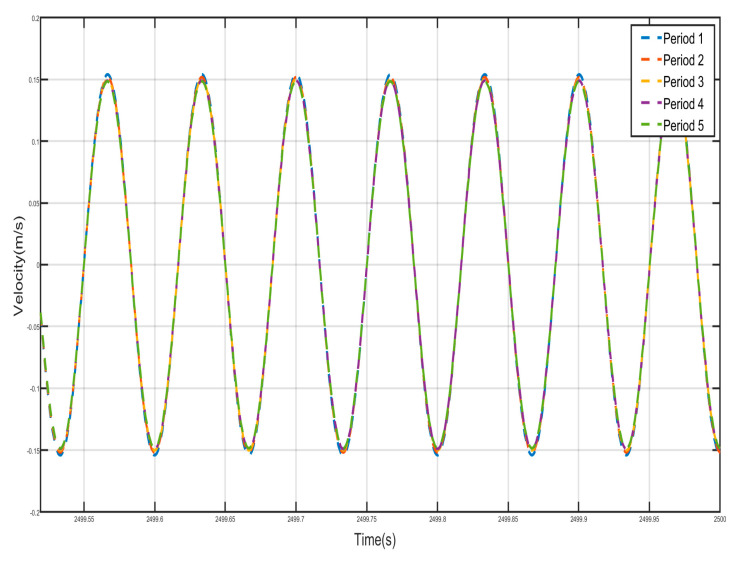
Five periodic shells’ velocity–time history curves. (zero mass).

**Figure 25:**
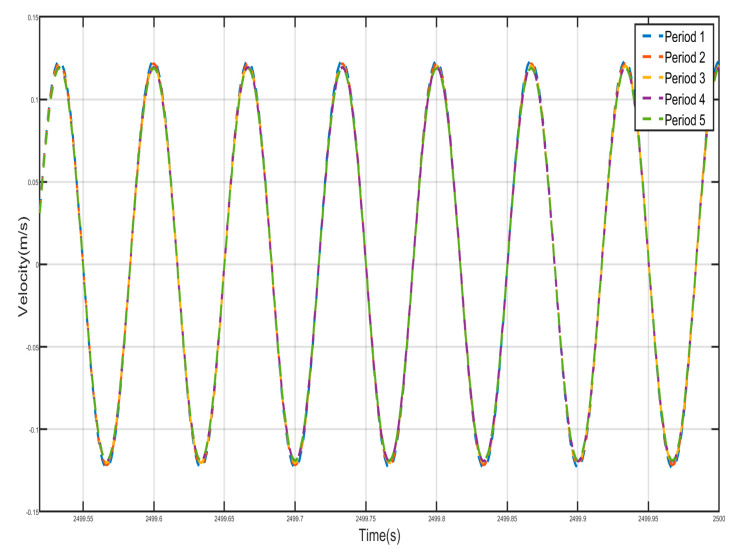
Five periodic negative-stiffness Duffing oscillators’ velocity–time history curves. (zero mass).

Figure 24 and Figure 25 show the velocity–time history curves of the shell and the negative-stiffness Duffing vibrator from the first period to the fifth period, from which it can be seen that the velocity–time history curves of the shells of the five periods are almost exactly overlapped, and the shells of all the period units move in the same-phase and same-velocity manner. The spring K connecting the periodic units does not deform, and the system reaches a stable state. If the motion of the internal Duffing oscillators is not considered, the entire system externally appears very much like a rigid rod, with the whole system always maintaining in-phase motion. At the same time, the velocity–time course curves of all internal negative-stiffness Duffing oscillators are almost exactly coincident, with no phase difference between them, and they are completely in the same direction and the same phase of vibration. However, for the shell and vibrator of the same period, the shell and vibrator synchronize but in reverse motion. At this time, the wave vector is zero, the wavelength of the system is infinite, so that there is no phase difference between the points within the system, and the displacement field of the system is always constant. The entire system exhibits translational motion. The effective negative mass system containing a Duffing oscillator with negative stiffness also exhibits zero mass.

## 4. Conclusions

This chapter is based on the theory of local resonance. For acoustic metamaterials containing weak nonlinear negative-stiffness Duffing oscillators, the perturbation method is used to obtain the first-order perturbation solutions for the acoustic frequency branch and the optical frequency branch, and then the bandgap starting and cutoff frequencies are obtained. The effects of the mass ratio and stiffness ratio on the bandgap starting and cutoff frequencies are analyzed, and the widening of the bandgap range relative to the positive-stiffness system is analyzed. Using numerical calculation methods, we verified how the bandgap starting frequency and cutoff frequency change with shell stiffness. We analyzed the effect of external excitation amplitude changes on the bandgap and discussed positive mass and negative mass phenomena within the bandgap range, as well as positive mass phenomena within the passband range and zero mass phenomena at the cutoff frequency. The numerical verification results were consistent with the theoretical analysis results. The following main conclusions are obtained:

(1) For an effective negative mass-periodic structure containing a negative-stiffness Duffing oscillator, the equations for its dimensionless dispersion curves are related to the coefficient $\bar{\Gamma }$ in front of the dimensionless nonlinear term, the steady-state amplitude of the negative-stiffness Duffing oscillator $\left|A_{2}^{\left(0\right)}\right|$, and the mass ratio β. When the local resonant oscillators are soft- and hard-characteristic negative-stiffness Duffing oscillators, respectively, their two dispersion curves shift toward the low- and high-frequency ranges relative to the linear system. Furthermore, under the same conditions, the influence of nonlinear factors on the optical frequency branch dispersion equation is greater than that on the acoustic frequency branch dispersion equation. The stronger the nonlinearity, the more pronounced this effect becomes.
(2) The bandgap starting frequency is related to the linear stiffness ratio (α) and the mass ratio (β). The starting frequency is lower than the resonance frequency, and it increases monotonically with the stiffness ratio (α) and decreases monotonically with the mass ratio (β). The bandgap cutoff frequency is related to the mass ratio (β), and it decreases monotonically with the mass ratio (β). The bandgap width in the negative-stiffness system exhibits approximately a $\sqrt{2}$-fold increase compared to its positive-stiffness system.
(3) The external excitation amplitude *F* changes the bandgap starting frequency and cutoff frequency, and as *F* increases, the starting frequency becomes higher and the cutoff frequency becomes lower. The bandgap width becomes small, and the corresponding bandgap frequency. When the external excitation amplitude is small, when the external excitation amplitude becomes large to a certain value, the vibration transmission characteristics of the system at the corresponding frequency have a tendency to decrease to increase, and are no longer located in the bandgap range.
(4) For an acoustic metamaterial containing a negative-stiffness Duffing oscillator, within its bandgap, the region between the resonant frequency and the cutoff frequency is a region of effective negative mass, and the region between the bandgap starting frequency and the resonant frequency is a region of effective positive mass. In the bandgap effective negative mass region, the motion of the negative-stiffness Duffing oscillator is in anti-phase with the motion of the shell and the propagation of vibrations is suppressed, while in the bandgap effective positive mass region, the motion of the negative-stiffness Duffing oscillator is in-phase with the motion of the shell but the propagation of vibrations is suppressed as well.
(5) The phenomenon of zero-mass also exists in the effective negative-mass structure containing a weakly nonlinear negative-stiffness Duffing oscillator, and the frequency point of zero-mass is the bandgap cutoff frequency.

## Figures and Tables

**Table 1:** Performance Comparison: Positive- and Negative-Stiffness Systems.

Name	Positive-Stiffness System	Negative-Stiffness System
Frequency at the Starting and Cutoff Point of the Dispersion Curve Bandgap Width (Hz)	0.9–1.11 5.67–7.67	1.2–1.6 7.9–10.8

## Data Availability

The original contributions presented in this study are included in the article material. Further inquiries can be directed to the corresponding authors.
